# Lactate Alters Metabolism in Human Macrophages and Improves Their Ability to Kill *Mycobacterium tuberculosis*

**DOI:** 10.3389/fimmu.2021.663695

**Published:** 2021-10-06

**Authors:** Cilian Ó Maoldomhnaigh, Donal J. Cox, James J. Phelan, Morgane Mitermite, Dearbhla M. Murphy, Gina Leisching, Lorraine Thong, Seónadh M. O’Leary, Karl M. Gogan, Kate McQuaid, Amy M. Coleman, Stephen V. Gordon, Sharee A. Basdeo, Joseph Keane

**Affiliations:** ^1^ TB Immunology Group, Department of Clinical Medicine, Trinity Translational Medicine Institute, St James’s Hospital, Trinity College Dublin, The University of Dublin, Dublin, Ireland; ^2^ School of Veterinary Medicine and Conway Institute, University College Dublin, Dublin, Ireland

**Keywords:** lactate, human, macrophage, glycolysis, metabolism, tuberculosis, seahorse, cytokine

## Abstract

In order to mount an appropriate immune response to infection, the macrophage must alter its metabolism by increasing aerobic glycolysis and concomitantly decreasing oxidative phosphorylation; a process known as the Warburg effect. Consequently, lactate, the end-product of glycolysis, accumulates in the extracellular environment. The subsequent effect of lactate on surrounding macrophages is poorly understood. *Mycobacterium tuberculosis* (Mtb), the causative organism of Tuberculosis (TB), is phagocytosed by macrophages in the airways. Mtb infected macrophages upregulate aerobic glycolysis and effector functions to try to kill the bacteria. Our lab has previously shown that human macrophages produce lactate in response to infection with Mtb. Although lactate has largely been considered a waste product of aerobic glycolysis, we hypothesised that the presence of extracellular lactate would impact subsequent immunometabolic responses and modulate macrophage function. We demonstrate that the presence of exogenous lactate has an immediate effect on the cellular metabolism of resting human macrophages; causing a decrease in extracellular acidification rate (ECAR; analogous to the rate of glycolysis) and an increase in the oxygen consumption rate (OCR; analogous to oxidative phosphorylation). When lactate-treated macrophages were stimulated with Mtb or LPS, glycolysis proceeds to increase immediately upon stimulation but oxidative phosphorylation remains stable compared with untreated cells that display a decrease in OCR. This resulted in a significantly reduced ECAR/OCR ratio early in response to stimulation. Since altered metabolism is intrinsically linked to macrophage function, we examined the effect of lactate on macrophage cytokine production and ability to kill Mtb. Lactate significantly reduced the concentrations of TNF and IL-1β produced by human macrophages in response to Mtb but did not alter IL-10 and IL-6 production. In addition, lactate significantly improved bacillary clearance in human macrophages infected with Mtb, through a mechanism that is, at least in part, mediated by promoting autophagy. These data indicate that lactate, the product of glycolysis, has a negative feedback effect on macrophages resulting in an attenuated glycolytic shift upon subsequent stimulation and reduced pro-inflammatory cytokine production. Interestingly, this pro-resolution effect of lactate is associated with increased capacity to kill Mtb.

## Introduction

Macrophage activation and effector function depends on a metabolic switch that results in increased flux through glycolysis and reduced oxidative phosphorylation; akin to the Warburg effect observed in cancer cells (1). Increased glycolysis enables macrophages to ramp up the production of cellular building blocks required for rapid proliferation and effector function including the production of pro-inflammatory IL-1β. Lactate, the end-product of glycolysis, accumulates as a consequence of this altered metabolic function (2–5). In keeping with this finding, assays that measure extracellular lactate concentrations have been used to indicate that this metabolic shift has taken place in activated macrophages (2–5). Lactate, for decades regarded as an unwanted waste product, has now been shown to have numerous functions, including as a carbon source, signaling molecule, histone deacetylase inhibitor (6) and, in the context of cancer, it influences cell growth and migration (7–9). Because many other types of immune cells also rely on this glycolytic switch for their activation, lactate is known to accumulate at sites of chronic inflammation and can propagate inappropriate inflammation *via* metabolic reprogramming (10). Clinically, elevated serum lactate levels are associated with increased mortality in a diverse range of patients (11, 12). Lactate levels also predict mortality in patients with infection (13) and sepsis (14).

Mycobacterium tuberculosis (Mtb), the bacteria that causes Tuberculosis (TB) disease, remains a significant global health burden; ranking as the single biggest infectious killer in 2019 with 1.4 million deaths estimated worldwide (15). A shift towards aerobic glycolysis, coupled with increased lactate production, takes place in human macrophages that are infected with Mtb (3–5, 16, 17). Furthermore, this increased glycolytic flux is associated with the ability of the macrophage to effectively kill Mtb (3–5), as inhibition of glycolysis in macrophages results in a reduced ability to kill Mtb (3). However, Mtb has evolved mechanisms to subvert the host immune response through reprogramming macrophage metabolism resulting in reduced lactate and IL-1β production (4, 5). These data underscore the importance of immunometabolism in host defence against Mtb. We postulated that an accumulation of lactate in the extracellular milieu, as a result of increased flux through glycolysis, may have a net benefit in host defence.

Infection with Mtb occurs after inhaled aerosolized droplets are phagocytosed by tissue resident alveolar macrophages (AM). AM are thought to be less effective at undergoing this shift to aerobic glycolysis than infiltrating monocyte derived macrophages (MDM) (18, 19) and the bacteria can live and replicate inside the AM, causing active TB disease. However, most people exposed to Mtb mount a sufficient immune response, whilst only a small proportion develop active TB.

Mtb stimulates a robust shift towards aerobic glycolysis leading to pro-inflammatory effector function in conjunction with increased production and secretion of lactate (3). While Mtb infected macrophages have been shown to alter the metabolic profiles of macrophages in their vicinity (20), a knowledge gap exists around what effect lactate accumulation specifically has on bystander macrophages that subsequently become infected if the initially infected macrophage fails to control the infection and the bacteria escape back into the extracellular space.

Lactate has been shown to inhibit glycolysis and cytokine production in human monocytes (2, 21) and murine macrophages stimulated with lipopolysaccharide (LPS) (22). Consistent with this, we hypothesised that exogenous lactate would downregulate cellular energetics in human macrophages infected with Mtb. Since cellular energetics are intrinsically linked to immune function, we hypothesised this altered immunometabolic phenotype would downregulate the pro-inflammatory function of the macrophage.

We demonstrate that lactate has an immediate effect on cellular metabolism, causing a rapid decrease in glycolysis (as marked by a drop in the extracellular acidification rate; ECAR) and an increase in oxidative phosphorylation (marked by the oxygen consumption rate; OCR). This altered metabolic phenotype induced in resting macrophages by the presence of lactate affects subsequent responses to Mtb or LPS by inhibiting the decrease in oxidative phosphorylation, thereby blocking the early Warburg effect. Lactate significantly reduced IL-1β and TNF production by human macrophages stimulated with Mtb, however, it also improved macrophage ability to kill Mtb. These data indicate that lactate has a potent immunomodulatory effect by downregulating subsequent macrophage activation, promoting resolution and aiding bacillary clearance.

## Materials And Methods

### MDM Cell Culture

Peripheral blood mononuclear cells (PBMC) were isolated from the buffy coats of healthy donors, obtained with consent from the Irish Blood Transfusion Services, by density-gradient centrifugation over Lymphoprep (StemCell Technologies). Cells were washed, resuspended at 2.5x10^6^ PBMC/ml in RPMI (Gibco) supplemented with 10% AB human serum (Sigma-Aldrich) and plated on to non-treated tissue culture plates (Costar). Cells were maintained in humidified incubators for 7 days at 37°C and 5% CO_2_. Non-adherent cells were removed by washing every 2-3 days. The purities of MDM were assessed by flow cytometry and were routinely > 90% pure.

### MDM Stimulation

On day 7 of differentiation, MDM were washed and treated with sodium L-lactate (25 mM, unless otherwise stated; Sigma Aldrich) or equimolar NaCl (Sigma Aldrich) or left untreated as a control. MDM were stimulated 3 hours post lactate treatment with LPS (Sigma-Aldrich; 100 ng/ml) or irradiated H37Rv (iH37Rv; gifted by BEI Resources). Macrophages were infected with a multiplicity of infection (MOI) of 1-10 bacteria per cell (approximately 70% infected). Briefly, cryopreserved iH37Rv was thawed, sonicated and passed through a 25-gauge needle prior to adding it to cells. Donor variation in phagocytosis of Mtb was adjusted for by Modified Auramine O (Scientific Device Laboratory), as previously described (23). For the experiments where analysis was undertaken after 24 hours, the extracellular bacteria were washed off after 3 hours, pelleted by centrifugation and half of the volume of supernatant was placed back on the well. Macrophages were then incubated for a further 21 hours in humidified incubators at 37°C and 5% CO_2_. Unstimulated macrophages were assayed in parallel as controls. MOI was also undertaken for the live analysis of Mtb stimulation in the Seahorse XFe24 Analyzer with the calculated volume of Mtb added to the ports of the Seahorse XFe24 Analyzer. Uninfected macrophages (sham injection of medium added through the ports) were assayed in parallel as controls.

To assess the impact of lactate (6.25-100mM; Sigma-Aldrich) on the viability of human MDM, cells were stained with 5 µg/mL propidium iodide (PI; Sigma-Aldrich), 20 µg/mL Hoechst 33342 (Sigma-Aldrich) and 50 µg/mL Hoechst 33258 (Sigma-Aldrich) at the indicated time-points post-stimulation with Mtb or LPS (100 ng/ml). Cells were incubated for 30 min at room temperature in the dark and analysed on the Cytell Cell Imaging System (GE Healthcare Life Sciences).

### Metabolic Assays Using the Seahorse XFe Analyzer

Macrophage metabolic function was assessed using the Seahorse XFe Analyzer (Agilent). After 7 days of adherence purification in the presence of 10% human serum, the medium was replaced with ice-cold PBS and the MDM placed at 4°C for 30 minutes. The MDM were then gently scraped and live cells were counted using trypan blue prior to re-plating onto Seahorse plates (1x10^5^ cells per well). The extracellular acidification rate (ECAR) and the oxygen consumption rate (OCR), surrogates for glycolysis and oxidative phosphorylation respectively, were measured 3 times every 10 minutes to establish baseline rates. For the live experiments the Seahorse Analyzer injected Seahorse medium or sodium L-lactate (25 mM; Sigma Aldrich) or equimolar NaCl dissolved in medium into assigned wells after 30 minutes. The ECAR and OCR readings were then continually sampled every 20 minutes in real time for 8 hours. Percentage change was calculated *versus* the third baseline readings. For the experiments with live analysis of Mtb or LPS, they were added to the assigned wells 3 hours after the lactate was added. For the later timepoint, MDM were stimulated with Mtb or LPS 24 hours prior to analysis in the Seahorse Analyzer. The Mito Flex Fuel Test (Agilent) was used to determine the dependency of human MDM on glucose, glutamine or fatty acid to maintain baseline respiration, according to the manufacturer’s protocol.

### Cytokine Production by ELISA

The concentrations of IL-1β, IL-10, TNF and IL-6 present in the supernatants were quantified using Meso Scale Discovery (MSD, Rockville, MD), according to the manufacturer’s protocol.

### Expression of Cell Surface Markers by Flow Cytometry

MDM were placed in ice cold PBS and incubated at 4°C on ice for 30 minutes. Cells were removed from the plastic by gentle scraping, Fc blocked with Human TruStain FcX (BioLegend) and stained with zombie NIR viability dye and fluorochrome-conjugated antibodies specific for CD14 (FITC), CD68 (PE), CD83 (PerCP-Cy5.5), CD80 (PE-Cy7), CD86 (BV410), CD40 (BV510), and HLA-DR or MMR (APC; all BioLegend). Cells were fixed with 2% PFA and acquired on a BD FACS Canto II. Unstained cells and FMO controls were used to normalise for background staining and to set gates. Data were analysed using FlowJo software.

### Mycobacterial Culture and Colony Forming Units (CFU)

Mtb H37Ra and H37Rv were obtained from The American Type Culture Collection (ATCC 25177TM; Manassas, VA) and propagated in Middlebrook 7H9 medium supplemented with ADC (Beckton Dickinson), to log phase. The multiplicity of infection (MOI) and donor variation in phagocytosis of Mtb was adjusted for by Auramine O staining, as previously described (23). To enumerate CFU, MDM were infected with a low MOI of 1-5 bacteria per cell and 30-40% infectivity. Na-L-Lactate (6.25 - 25mM; Sigma Aldrich) or equimolar NaCl was added 3 hours prior to infection. Extracellular bacteria were washed off 3 hours post infection and macrophages were incubated as indicated. CFU were determined at day 0 (3h post-infection), 2, or 5, as indicated; cells were lysed with Triton X-100 (0.1%) and pooled with bacterial pellets (at all timepoints except day 0) from the centrifugation of supernatants. Bacteria were diluted in Middlebrook 7H9 broth and plated onto Middlebrook 7H10 agar supplemented with OADC (both Becton Dickinson) and cycloheximide (Sigma-Aldrich). CFU counts were performed 21-days post incubation at 37°C.

### Autophagy Assays

MDM were treated with lactate (25 mM) for two hours prior to the addition of the autophagy inhibitor 3MA (5 µM; Merck). Cells were incubated for a further hour prior to the addition of Mtb (H37Ra; MOI 1-10 bacteria/cell, 70% infectivity). 3 hours post infection, cells were washed to remove any remaining extracellular bacteria which was pelleted by centrifugation. Supernatants were added back to the wells and cells were maintained in humidified incubators for 24 hours post infection at 37°C and 5% CO_2_. Western blot analyses was undertaken to characterise expression levels of LC3 and p62, proteins involved in autophagy. 24 hours post infection with Mtb, MDM were washed with PBS and lysed in lysis buffer (4:2:1:1 H20/10% SDS/glycerol/0.5 M Tris, pH 6.8 containing Dichlorodiphenyltrichloroethane (10%) with the addition of protease (ThermoFisher Scientific) and phosphatase inhibitor tablets (Sigma Aldrich). MDM were then scraped and the protein lysate harvested and immediately heated to 95°C to inactivate proteases. Sodium dodecyl sulfate (SDS) sample buffer (100mM Tris-HCl (pH 6.8), 20% Glycerol (v/v), 4% SDS (w/v), 0.001% bromophenol blue (w/v) containing 143 mM dithiothreitol) was added to the lysates and equal volumes of the lysates (30 μl/lane) were resolved by SDS polyacrylamide gel electrophoresis (SDS-PAGE) (10%) using the Bis-Tris Electrophoresis System (Bio-Ray). The transfer of separated proteins to polyvinylidene fluoride membrane was performed by wet blotting (Bio-Rad). The PVDF membranes were blocked with blocking buffer containing 5% milk (Marvel) in Tris- Buffered saline Tween (TBST) (0.1% (v/v) Tween-20 in TBS) at room temperature for 1 hour. Following blocking of the membrane, the immunoblot was incubated with purified mouse anti-human LC3 (1:1,000), purified rabbit anti-human p62 (1:1,000) or purified mouse anti-human Actin (1:1,000) at 4°C overnight in TBST (Nanotools). Followed by incubation with secondary goat anti-mouse or anti-rabbit IgG peroxidase conjugated antibody (Millipore) diluted in TBS for 1 hour at room temperature. The immunoblots were developed using enhanced chemiluminescence (ECL; MyBio) and visualised using a chemiluminescence imaging system (Fusion FX).

To determine if the bactericidal effects of lactate were mechanistically linked to promoting autophagy, MDM were infected with H37Ra at an MOI of 1-5 and 30-40% infectivity in the presence or absence of 3MA and lactate. Cells were lysed on day 0, 2 and 5 and CFU were enumerated as outlined above.

### Statistical Analysis

Statistical analyses were performed using GraphPad Prism 9 software. Statistically significant differences between two normally distributed groups were determined using Student’s paired t-tests with two-tailed *P*-values. Differences between three or more groups were determined by one-way ANOVA with Tukey’s multiple comparisons tests. Differences between two or more groups containing more than one variable were determined by two-way ANOVA with Sidak’s multiple comparisons tests. *P*-values of <0.05 were considered statistically significant and denoted using an asterisk.

## Results

### Lactate Decreased ECAR and Increased OCR in Human MDM

Lactate has previously been shown to alter the metabolism of human monocytes one hour after treatment (2). We sought to determine if exposure to exogenous lactate similarly affects the metabolic function of resting human MDM in real time.

Physiological levels of lactate in human peripheral blood range between 0.5-2 mM, however, this increases in disease states such as sepsis and have been reported as high as 25 mM after strenuous exercise (24, 25). Lactate is released from lung tissue in patients with sepsis and acute respiratory distress syndrome (26). Local tissue concentrations of lactate during acute infections or in the context of chronic inflammation are largely unknown but concentrations of approximately 15 mM have been measured in the synovial joints of patients with rheumatoid arthritis (27). Furthermore, published work reports macrophages stimulated *in vitro* have varying concentrations of lactate in the supernatants, up to approximately 30 mM (5).

Cell viability was assessed to determine the optimal concentration of lactate to treat MDM with *in vitro*. MDM were treated with a range of lactate concentrations (6.25-100 mM) prior to stimulation with LPS or iH37Rv Mtb or infection with H37Ra Mtb. Cell viability was then examined 24, 48 and 120 hours post stimulation (**Supplementary Figure 1**). 25 mM lactate was the highest concentration that did not induce statistically significant cell death at any time point, with or without stimulation (**Supplementary Figure 1**) and, as this concentration was physiologically relevant, it was used in subsequent experiments.

There has been contradictory evidence over the necessity of acidification in order to see a cellular effect of lactate (21, 28–30). We used sodium-L-lactate which did not affect the pH of the medium at any concentration between 6.25 and 100 mM (data not shown). Moreover, any disturbances in pH would be reflected in the metabolic Seahorse analyses, which was not observed in our model. To control for potential osmotic stress and any potential metabolic effects caused by the presence of the sodium ion, we used sodium chloride (31, 32) at an equimolar concentration as a control (**Supplementary Figure 2**).

**Figure 1:**
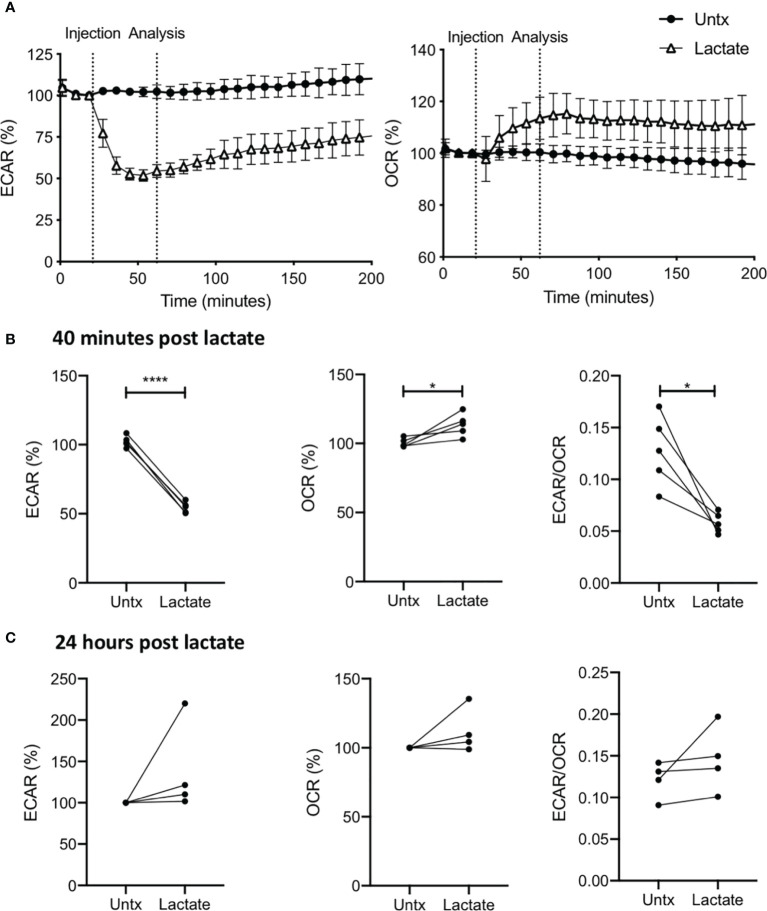
Lactate induces metabolic changes in human MDM. PBMC were isolated from buffy coats and MDM were adherence purified for 7 days in 10% human serum. MDM were gently scraped and seeded on Seahorse culture plates prior to analysis in the Seahorse XFe24 Analyzer. Lactate (25 mM) was either added to the cells through the Seahorse Analyzer ports **(A, B)** or 24 hours prior to analysis **(C)**. Correction for differences in cell density was achieved by % comparison to the basal ECAR and OCR for the real-time analysis and by % comparison to the untreated MDM after crystal violet normalization for the analysis 24 hours post treatment. The ECAR and OCR were recorded approximately every 9 minutes. After 30 minutes, the SeahorseXFe24 Analyzer injected lactate or control medium (untreated; untx) into assigned wells. The ECAR and OCR readings were then continually sampled in real time. The time-course graphs illustrate the ECAR and OCR **(A)** of human MDM in response to treatment with lactate. At the time point indicated, 40 mins after administration, the effect of lactate on ECAR, OCR and the ECAR/OCR ratio **(B)** were calculated (n=5 ± SD). 24 hours after treatment with lactate, the basal ECAR, OCR and the ECAR/OCR ratio **(C)** were calculated (n=4 ± SD). Statistical significance was determined using a paired t test; *P < 0.05, ****P < 0.0001.

We performed a real-time kinetic analysis of the metabolic effect of exogenous lactate (25 mM) on human MDM using the Seahorse XFe24 Analyzer. MDM were monitored for 30 minutes before lactate or medium (untreated control) was injected into wells through the port in the Seahorse Analyzer. The ECAR and OCR is expressed as % change from this baseline in order to correct for human variability and minor differences in cell seeding density on the Seahorse culture plate. Addition of lactate to resting (unstimulated) human MDM caused an immediate decrease in ECAR and increase in OCR (**Figure 1A**) compared with untreated (denoted as “untx”) controls. Analysis at 70 mins (40 mins post treatment with lactate) collated from n=5 independent experiments showed significantly decreased ECAR (P<0.001), increased OCR (P<0.05) and reduction in the ECAR/OCR ratio (P<0.05; **Figure 1B**). No significant differences were seen in the NaCl control (**Supplementary Figures 2A, B**). 24 hours post the addition of lactate, no significant differences were observed in the ECAR, OCR or the ECAR/OCR ratio compared to untreated MDM (**Figure 1C**).

### Pre-Treatment With Lactate Changes the Immunometabolic Response of Human MDM to LPS and Mtb Stimulation

Having established that exogenous lactate has an immediate effect on the metabolic function of resting macrophages, we sought to determine how this would affect their ability to shift metabolic function towards glycolysis upon stimulation with Mtb or LPS. This scenario models the environmental milieu of early infection events, with bystander uninfected macrophages exposed to increasing levels of lactate produced by neighbouring infected cells that have increased glycolysis.

**Figure 2:**
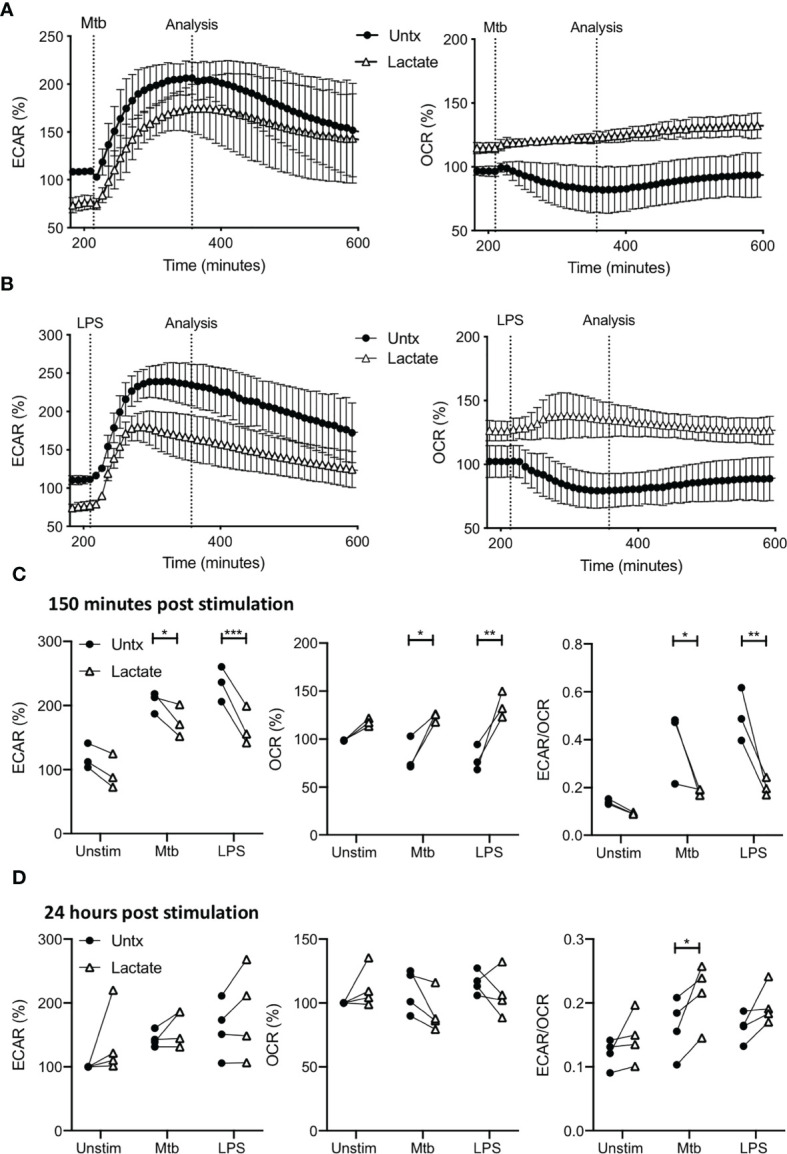
Pre-treatment with lactate changes the immunometabolic response of human MDM to Mtb or LPS stimulation. PBMC were isolated from buffy coats and MDM were adherence purified for 7 days in 10% human serum. MDM were gently scraped and seeded on Seahorse culture plates prior to analysis in the Seahorse XFe24 Analyzer. Lactate (25 mM) was added 3 hours prior to stimulation with Mtb (iH37Rv; MOI 1-10) or LPS (100 ng/ml). Mtb or LPS were either added to the cells in the Seahorse Analyzer (**A–C**; n=3 ± SD) or 24 hours prior to analysis (**D**; n=4). Correction for differences in cell density was achieved by % comparison to the basal ECAR and OCR for the real-time analysis and by % comparison to the lactate untreated MDM after crystal violet normalization for the analysis after 24 hours. The time-course graphs illustrate the ECAR and OCR of human MDM in real-time in response to stimulation with Mtb **(A)** or LPS **(B)** 3 hours after lactate was injected. At the time point indicated, approximately 150 minutes after stimulation, the effects of lactate on ECAR, OCR and the ECAR/OCR ratio **(C)** were calculated. Human MDM that have been pre-treated with lactate for 3 hours and then stimulated with either Mtb or LPS, were analysed in the Seahorse Analyzer 24 hours after stimulation. The basal ECAR, OCR and ECAR/OCR ratio **(D)** are shown. Statistical significance was determined using two-way ANOVA with Sidak’s multiple comparison test; *P < 0.05, **P < 0.01, ***P < 0.001.

MDM were monitored for 30 minutes prior to the addition of lactate as in **Figure 1**. 3 hours post the addition of lactate, MDM were stimulated with Mtb (iH37Rv) or LPS by injection through the ports of the SeahorseXFe24 Analyzer. The kinetics of the ensuing responses were monitored by analyzing the ECAR and OCR in real time every 9 minutes as indicated in **Figure 2**. The time-course graphs represent the ECAR and OCR of MDM treated with lactate for 3 hours (graphs begin at approximately 200 minutes from the start of the experiment) then stimulated with Mtb (**Figure 2A**) or LPS (**Figure 2B**); data collated from 3 independent experiments.

Due to the early effects of lactate, the ECAR is lower and OCR is higher compared with untreated controls prior to stimulation with Mtb or LPS, as shown in the timepoints up to 200 minutes (**Figure 1**). The presence of lactate does not affect the rate of increase in ECAR in response to either Mtb or LPS (**Figures 2A, B**, left), however, since the ECAR starts from a lower baseline due the presence of lactate, its plateau at 150 minutes post stimulation is significantly reduced compared with untreated macrophages stimulated with Mtb (P<0.05) or LPS (P<0.001; **Figure 2C**, left).

Conversely, the OCR is elevated in MDM pre-treated with lactate and remains stable upon stimulation with either Mtb or LPS compared with untreated MDM which decrease OCR in response to stimulation (**Figures 2A, B**, right). Collated data from the analysis timepoint approximately 150 minutes post stimulation (approximately 350 minutes from the outset of the experiment), shows that lactate significantly increased the OCR of MDM stimulated with Mtb (P<0.05) or LPS compared with untreated controls (P<0.01; **Figure 2C**, middle). This decrease in ECAR and concomitant increase in OCR at 150 minutes post stimulation results in a statistically significantly reduced ECAR/OCR ratio in lactate treated MDM following Mtb (P<0.05) or LPS (P<0.01) stimulation compared with untreated controls (**Figure 2C**, right).

MDM treated with or without lactate were assessed 24 hours post stimulation with Mtb or LPS (**Figure 2D**). Correction for differences in cell density was achieved by % comparison to the basal ECAR and OCR of the unstimulated, untreated MDM after crystal violet normalization. Lactate did not significantly alter the ECAR or OCR of MDM stimulated with Mtb or LPS compared with untreated controls. However, the ECAR/OCR ratio for lactate-treated MDM stimulated with Mtb was significantly increased compared with untreated controls (P<0.05; **Figure 2D**, right).

In order to determine if lactate alters other metabolic pathways in human MDM, we used the Mito Flex Fuel test to measure the cellular dependency on glucose, glutamine or fatty acids in cells treated with lactate for 24 hours *versus* untreated controls, and in the context of stimulation with Mtb (iH37Rv; **Supplementary Figure 3**). Lactate did not significantly alter glutamine or glucose dependency but significantly increased the dependency on fatty acids (P<0.01; **Supplementary Figure 3**) in resting macrophages compared with untreated controls.

In summary, we have shown that lactate causes an immediate decrease in ECAR and increase in OCR in resting human MDM suggesting that lactate tempers glycolysis in surrounding macrophages. Upon stimulation, the presence of lactate prevents the reduction in oxidative phosphorylation (thereby preventing the Warburg effect) in human MDM early in their response to Mtb or LPS. The glycolytic shift required for MDM activation is intact, albeit diminished. This resulted in a significantly decreased ECAR/OCR ratio in stimulated MDM treated with lactate. At the later timepoint, 24 hours post stimulation, MDM treated with lactate do not exhibit significantly different ECAR or OCR compared with untreated controls, however, the presence of lactate significantly increased the ECAR/OCR ratio in Mtb stimulated MDM. In addition, lactate increased the dependency of resting human MDM on fatty acids to maintain baseline respiration.

These data indicate that the presence of exogenous lactate downregulates glycolytic metabolism and promotes oxidative phosphorylation. Whilst lactate allows for a shift towards glycolysis to occur if the macrophage becomes subsequently stimulated, this shift is attenuated compared with control. This suggests that lactate, the end-product of glycolysis, exhibits an inhibitory, negative feedback type role by reducing glycolysis and may therefore, reduce macrophage activation, inflammation and downstream effector functions.

### The Presence of Lactate Reduces Cytokine Production in MDM Stimulated With Mtb or LPS

Since our data indicated that lactate reduced glycolysis and blocked the Warburg effect in human macrophages stimulated with Mtb, and since glycolytic function is closely associated with pro-inflammatory function (3, 4, 33–37), we sought to determine if lactate modulated cytokine production in human macrophages. We treated MDM with lactate for 3 hours prior to the addition of Mtb or LPS. The concentrations of IL-1β, TNF, IL-10 and IL-6 present in the supernatants were quantified by Meso Scale Discovery system ELISA.

**Figure 3:**
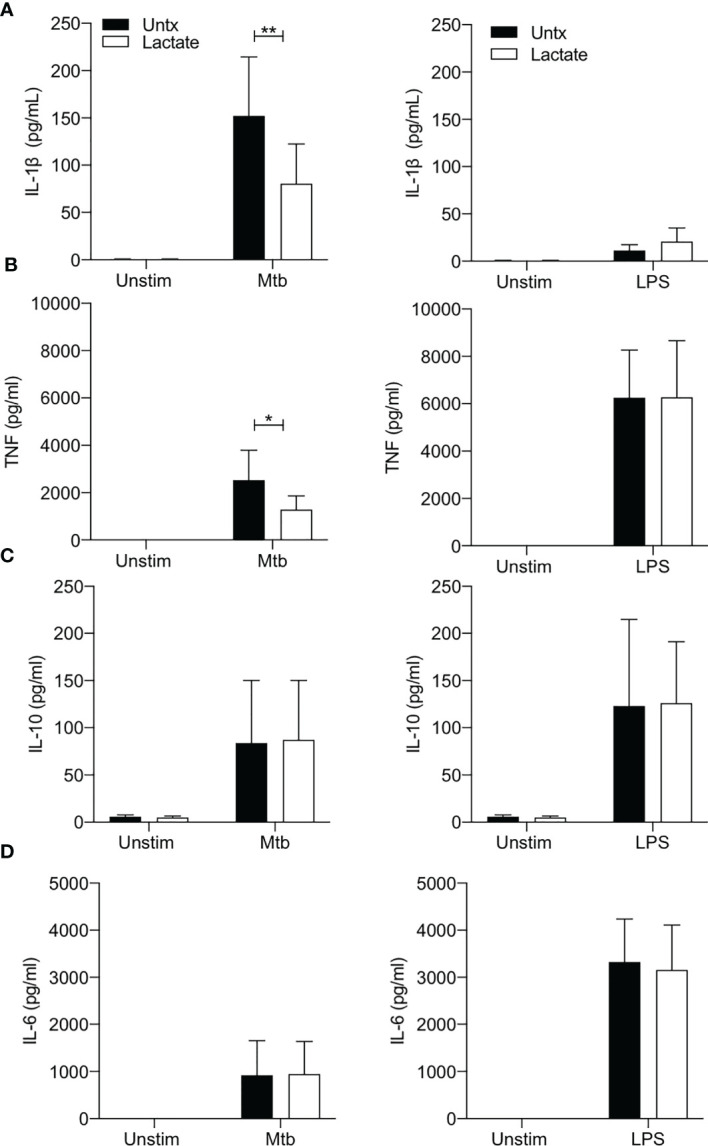
Lactate reduces TNF and IL-1β production. PBMC were isolated from buffy coats and MDM were adherence purified for 7 days in 10% human serum. Cells were treated with lactate (25 mM) for 3 hours prior to stimulation with Mtb (iH37Rv; MOI 1-10) or LPS (100 ng/ml). The concentrations of IL-1β **(A)**, TNF **(B)**, IL-10 **(C)** and IL-6 **(D)** in supernatants were measured by Mesoscale Discovery assay 24 hours after stimulation with Mtb or LPS (n=4). Statistical significance was determined using two-way ANOVA with Sidak’s multiple comparison test; *P < 0.05, **P < 0.01.

The production of IL-1β is closely associated with increased glycolysis and accompanying changes in cellular energetics in murine macrophages stimulated with LPS (33). In addition, we have shown that increased glycolysis in the context of Mtb infection is associated with increased IL-1β production (3, 4, 34, 35). In keeping with the observation that lactate attenuated glycolysis in macrophages stimulated with Mtb, our data shows significantly reduced concentrations of IL-1β present in the supernatants of lactate-treated macrophages 24 hours post stimulation with Mtb (P<0.01; **Figure 3A**). No differences were observed between lactate-treated and untreated MDM stimulated with LPS, as expected since there is no second signal to activate the inflammasome to produce mature IL-1β.

TNF is a key proinflammatory cytokine produced by activated macrophages and is an important factor in the host response to Mtb (38, 39). Lactate has previously been shown to modulate TNF production in human cells stimulated with LPS (2, 21, 29). We therefore sought to determine if lactate affected TNF production in human MDM stimulated with Mtb or LPS (**Figure 3B**). Lactate significantly reduced TNF production in MDM stimulated with Mtb (P<0.05) but did not affect LPS induced TNF production (**Figure 3B**).

The concentrations of IL-10 (an anti-inflammatory cytokine) or IL-6 (a key pyrogenic cytokine) produced in response to stimulation with either Mtb or LPS were not significantly affected by lactate (**Figures 3C, D**).

We sought to determine if lactate had an impact on the expression of cell surface markers associated with antigen presentation and “M1/M2” phenotype. We examined the expression of HLA-DR, CD40, CD80, CD86, CD83 and the mannose receptor (MMR) by flow cytometry in human MDM treated with lactate compared with controls, and under conditions of stimulation with Mtb or LPS. Lactate did not significantly alter the cell surface expression of these markers (**Supplementary Figure 4**).

### Lactate Improves Human MDM Killing of Mtb

**Figure 4:**
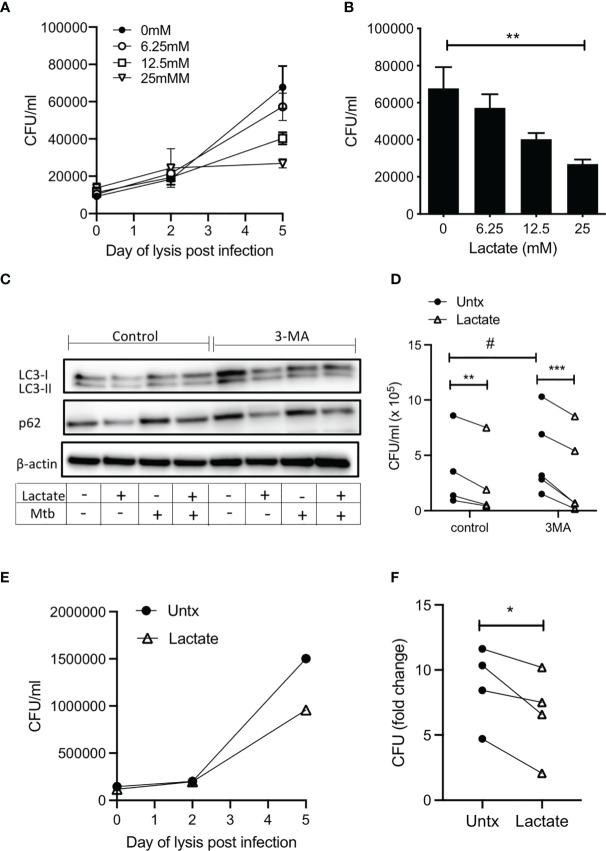
Lactate improves human MDM killing of Mtb by promoting autophagy. PBMC were isolated from buffy coats and MDM were adherence purified for 7 days in 10% human serum. **(A–D)** MDM were left untreated or treated with 6.25, 12.5 or 25 mM of lactate for three hours prior to infection with Mtb (H37Ra) at a MOI of 30-40% infectivity, 1-5 mycobacteria per cell (as defined by auramine O staining) and were then lysed on day 0 (3 hours post-infection), day 2 and day 5 and plated on Middlebrook 7H10 agar (+OADC). CFU were enumerated 21 days after plating. The time course graph shows the CFU results at day 0, 3 and 5 for each of the lactate concentrations **(A)**; n=5± SD). The differences in CFU growth for each concertation of lactate at the day 5 time point are graphed in **(B)** (n=5 ± SD). Cells were treated with the autophagy inhibitor, 3MA (5 µM), in the presence or absence of lactate (25 mM) 1 hour prior to infection with H37Ra. Cells were lysed 24 hours post infection and the expression of LC3-I, LC3-II, p62 and β-actin were determined by Western Blotting **(C)**; representative blot of n=4 experiments). Cells were lysed 5 days post infection to enumerate CFU **(D)**; n=4. **(E, F)** MDM were treated with lactate (25 mM) for 3 hours prior to infection with H37Rv at a MOI of 30-40% infectivity, 1-5 mycobacteria per cell (as defined by auramine O staining) and were then lysed on day 0 (3 hours post-infection), day 2 and day 5 and plated on Middlebrook 7H10 agar (+OADC). CFU were enumerated 21 days after plating. Representative growth curve of H37Rv over time in untreated compared with lactate treated human MDM **(E)**. Collated data showing the fold change in CFU on day 5 relative to day 0 controls **(F)**; n=4. Statistical significance was determined using one-way or two-way ANOVA, as appropriate, with Tukey’s or Sidak’s comparison test; **P < 0.01, ***P < 0.001, or paired Student’s t-test; *P < 0.05, ^#^ *P*< 0.05.

We have previously reported increased endogenous lactate produced by human MDM in response to infection with Mtb (3, 4). Moreover, this shift towards aerobic glycolysis, coupled with increased lactate, was associated with bacillary killing. We sought to determine if lactate had a cell-mediated effect on bacterial growth. We used live Mtb H37Ra to examine the effect of lactate on the ability of MDM to control an infection. MDM were pretreated with a range of concentrations (6.25 – 25 mM) of lactate 3 hours prior to infection with Mtb H37Ra. MDM were lysed on day 0 (3 hours after infection), day 2 and day 5, plated onto Middlebrook agar supplemented with OADC, incubated for 21 days and colony forming units (CFU) were enumerated. Collated data from n=5 independent experiments indicates that the level of infection is similar in all groups on day 0 and by day 5, untreated macrophages exhibit increased bacterial growth (**Figure 4A**). Lactate caused a dose dependent reduction in CFU that were plated from MDM lysed 5 days after infection, with a statistically significant decrease in CFU in MDM treated with 25 mM lactate compared with untreated controls (P<0.01; n=5; **Figure 4B**). No differences in CFU growth were seen with NaCl at equivalent molar concentrations (**Supplementary Figure 2C**).

Autophagy is an essential protective strategy employed by the host, however, Mtb has been shown to disrupt autophagic flux (40). Lactate has been shown to increase autophagy in cancer cells (41) although the impact of lactate on autophagy in human MDM is unknown. LC3 and p62 are cytosolic proteins that are recruited to the autophagosome upon initiation of autophagy and are degraded upon fusion of the autophagosome with the lysosome. Accumulation of LC3 and p62 after initiation of autophagy is indicative of a block in autophagic flux. 3-methyladenine (3MA) is a phosphoinositide 3-kinase (PI3K) inhibitor that has been shown to block autophagy (42) and increase *Mycobacterium aurum* growth in a THP-1 cell line (43).

In order to test the hypothesis that induction of autophagy by lactate is the mechanism by which Mtb killing is increased, expression of LC3 and p62 in human MDM infected with Mtb (H37Ra) for 24 hours in the presence or absence of lactate and in the presence or absence of 3MA (5 µM) was determined using Western blotting. LC3-I is found in the cytoplasm and converts to the LC3-II which is lipidated and therefore can be membrane bound. This conversion promotes the recruitment of ubiquitinated p62, marking the autophagosomal complex for delivery to the lysosome and degradation (44). Our data shows that lactate reduced the expression of LC3-II protein levels in uninfected macrophages but does not alter LC3-II expression during Mtb infection. This could be explained by the fact that the uninfected/resting macrophages have lower basal levels of autophagy, thus with the addition of lactate, a slight, yet unsignificant decrease in LC3-II was observed. Accumulation of p62, indicative of a block on autophagy, is evident in Mtb infected and 3MA treated human MDM (**Figure 4C**). The addition of lactate reduced the expression of p62 in both resting and Mtb infected MDM and in MDM treated with 3MA (**Figure 4C**), indicating increased flux through the pathway. Densitometry data quantifying the ratio of LC3-II or p62 to β-actin are shown in **Supplementary Figure 5A** along with Western blot images from 3 other donors (**Supplementary Figures 5B, C**). On day 5 post infection, cells were lysed to enumerate CFU (**Figure 4D**). Blocking autophagy with 3MA significantly increased bacterial growth compared with control (P<0.05), however; lactate significantly reduced CFU in both untreated (P<0.01) and 3MA treated cells (P<0.001). Taken together, the evidence indicates that lactate promotes autophagy by reducing p62 expression in both resting and Mtb infected MDM and MDM treated with 3MA, which suggests that lactate mediates its bactericidal effects, at least in part, through enhancing autophagy.

In order to ensure that the effect of lactate on bacterial growth is recapitulated in the context of virulent Mtb, human MDM were treated with lactate for 3 hours prior to infection with H37Rv. MDM were lysed on day 0 (3 hours after infection), day 2 and day 5, plated onto Middlebrook agar supplemented with OADC, incubated for 21 days and colony forming units (CFU) were enumerated (**Figure 4E**). Fold change in CFU on day 5 relative to day 0 indicates that lactate significantly reduced the growth of virulent Mtb in human MDM compared with control (**Figure 4F**; P<0.05, n=4).

Having established that lactate exhibits profound effects on metabolic function; by inhibiting the early Warburg effect but elevating the ECAR/OCR ratio 24 hours post infection, we sought to determine if lactate altered metabolic function 5 days post infection, when the effects on bacterial growth are evident. However, lactate did not significantly alter the ECAR/OCR ratio of human MDM on day 5 post stimulation (**Supplementary Figure 5D**). Similarly, the effect of lactate on cytokine production was assessed at this later time point, however, the concentrations of cytokines detectable at day 5 were not significantly increased in Mtb stimulated cells compared with unstimulated controls and lactate did not significantly alter cytokine production (**Supplementary Figure 5E**).

## Discussion

We have shown that lactate has an immediate effect on human macrophage metabolism, reducing glycolysis and increasing oxidative phosphorylation, a reverse of the Warburg effect. This impacts subsequent immunometabolic responses and macrophage function including decreasing cytokine production and improving the ability of human macrophages to kill Mtb.

Previous studies have reported that lactate inhibits glycolysis in PBMC, monocytes and mast cells (2, 21, 29, 45). In keeping with these published findings, we have shown that lactate causes a rapid and significant reduction in glycolysis in differentiated human macrophages but does not prevent the glycolytic shift following stimulation with Mtb or LPS. While lactate reduces the peak of glycolysis following stimulation, this is due to lactate reducing the glycolytic rate prior to stimulation. Interestingly, lactate increased oxidative phosphorylation in resting macrophages and blocked the reduction in oxygen consumption observed early after stimulation with Mtb or LPS. Taken together, lactate attenuated glycolytic metabolism and concomitantly maintained oxygen consumption; in effect, blocking the early shift to Warburg metabolism observed in human macrophages immediately after stimulation. Our group are the first to report that human macrophages undergo early Warburg metabolism in response to Mtb or LPS stimulation (36) and the importance of this early metabolic switch in human immune cells is still poorly understood.

During an active macrophage infection, lactate accumulates in the extracellular environment due to increased glycolysis. Our data indicates that lactate then exerts a negative feedback type mechanism on neighbouring uninfected macrophages; inhibiting further Warburg metabolism but maintaining the ability to shift towards glycolytic metabolism, albeit attenuated. This suggests that the early decrease in oxidative phosphorylation may be a critical regulatory node in the activation of proinflammatory macrophage responses. *In vivo*, the negative effect of lactate on bystander metabolism may have multiple purposes including downregulating inflammation to limit collateral tissue damage, promoting resolution and a return to homeostasis, or may be a mechanism by which bystander macrophage glucose consumption is restricted to support the nutrient needs of neighbouring infected macrophages that are producing the lactate; in keeping with published observations showing that the metabolic function of one immune cell can be regulated by increased demand on nutrients from another immune cell in the environment (46).

Our group have previously demonstrated that conditioned medium (which contains lactate in addition to cytokines and other soluble factors) from taken from macrophages 24 hours post infection with Mtb promoted ECAR and reduced OCR in resting macrophages after 90 minutes of exposure to the conditioned medium (20). This suggest that soluble factors in conditioned medium from infected macrophages can promote Warburg metabolism in bystander macrophages whereas the data shown here demonstrates that lactate inhibits the Warburg effect. These seemingly incongruent findings suggest that lactate signalling may have to compete with other factors present in the environment during an infection, such as pro-inflammatory cytokines, to exert its downregulatory effects on glycolysis. Therefore, the ratio of lactate produced to other soluble factors may be critical in determining the bystander effect on metabolic function during infection. Our data indicates that timing may also be a contributing factor, as the downregulatory effects of lactate on glycolytic metabolism are mostly short-lived. Therefore, soluble factors in the environment produced early in response to infection may alter the metabolic phenotype of bystander macrophages in a different manner compared with other factors, such as mature secreted cytokines, that are produced later.

The production of mature IL-1β is linked to Warburg metabolism in mice (33) and to the ability of human macrophages to increase glycolysis in response to infection with Mtb (3, 4, 34, 35). We observed significantly reduced concentrations of IL-1β in Mtb-stimulated MDM pre-treated with lactate compared with control. This suggests that the maximal rate of glycolysis early in response to infection and not the degree of change, may be an important factor in the subsequent production of mature IL-1β. We also observed significantly reduced concentrations of TNF in Mtb-stimulated MDM pre-treated with lactate compared with control. Studies with murine macrophages *in vitro* show that modulating glycolysis has no effect on TNF production (33, 37) but inhibiting glycolysis in an *in vivo* mouse model of Mtb infection results in reduced TNF production from macrophages (18). We have previously shown in human macrophages that promoting glycolysis during Mtb infection resulted in increased TNF production (34, 35). Lactate bore a similar effect on the metabolic flux of macrophages stimulated with LPS compared to those stimulated with Mtb. In contrast, lactate did not reduce the concentrations of TNF produced by LPS stimulated macrophages. This is in keeping with published work showing that lactate did not reduce TNF production in LPS-stimulated human monocytes (2). These findings highlight stimulation-specific downstream effects of metabolic manipulation on macrophage effector function. Furthermore, umbilical cord blood macrophages fail to reduce oxidative phosphorylation upon stimulation, and also produce less TNF specifically in response to Mtb infection but not LPS-stimulation compared with adult macrophages (36). Cumulatively, these findings suggest that there may be a metabolic link to TNF production in macrophages in the context of Mtb infection that requires further elucidation.

Despite the reduction in pro-inflammatory cytokine production, lactate promoted Mtb killing in human MDM. Lactate can function as a carbon source for Mtb and thus promote bacterial growth but, at high concentrations, can inhibit growth in axenic conditions (47). Our data suggests that the effects of lactate on bacillary clearance are cell mediated because of the dose-dependent effect observed on bacterial growth. In addition, our infection model protocol removes extracellular bacteria 3 hours post infection by thoroughly washing and no differences are observed in the number of live bacteria phagocytosed by MDM during this timeframe (as evidenced by CFU counts at day 0 and confirmed by auramine O staining). Our mechanistic data indicates that lactate enhances bacterial clearance by promoting autophagy. The ability of lactate to promote autophagy may be independent of its effect on metabolic function, however these pathways are intrinsically linked because nutrient stress in the environment triggers autophagy and modulates metabolic function. Furthermore, both glycolysis and autophagy rely on mTOR signalling (41) indicating that these processes may be linked in macrophages (48).

Our data indicated that while lactate attenuated glycolysis and blocked the early Warburg effect, the ECAR/OCR ratio in macrophages infected with Mtb was significantly elevated 24 hours post stimulation with lactate which may result in increased capacity to kill bacteria. However, we did not find evidence that lactate could support glycolysis over oxidative phosphorylation at day 5 post infection.

### Study Limitations

Our work is the first, to our knowledge, to show that lactate reduced glycolysis and increased oxidative phosphorylation in fully differentiated human macrophages and that its presence blocked the early Warburg effect in macrophages subsequently stimulated with Mtb or LPS. The mechanism by which lactate mediates this metabolic effect remains unknown and is beyond the scope of this current work.

We have determined that lactate increased fatty acid dependency in resting MDM compared with controls, but this effect on fatty acid metabolism is not apparent 24 hours post stimulation with Mtb. Therefore, further work is required to fully elucidate the kinetics of the effects of lactate on fatty acid metabolism.

In addition, we do not have direct evidence linking the metabolic changes observed with the effects on cytokine production or CFU, however, there is ample rationale in the literature to propose that these effects are connected with altered metabolic function.

Our data suggests that lactate increased autophagy across all treatments, although in the presence of 3-MA, we did not observe an increase in LC3-II that was expected. We therefore cannot comment on flux in this particular model; however, these results can be explained by a study that found that 3-MA induces autophagy in some settings (42), which corresponds the with lower levels of LC3-II proteins and decreased p62 protein expression observed in this work. We therefore acknowledge this as a limitation in our study and in order to better characterize the effects that lactate may have on autophagic flux, bafilomycin may be a compound better suited for this purpose.

Since p62 was decreased by lactate, further work is warranted to explore the effects of lactate on reactive oxygen species, eicosanoids and leukotrienes.

Mtb has been shown to subvert macrophage metabolism (4, 49), therefore, we used irradiated H37Rv (iH37Rv) strain of Mtb to elucidate the host metabolic response in the presence of lactate, unperturbed by interference from live, growing Mtb. The effects of lactate on the ability of MDM to kill Mtb was performed with live, attenuated Mtb H37Ra to model the successful host immune response and was recapitulated in a model using live, virulent Mtb H37Rv. We acknowledge that further study in clinical strains is required.

## Conclusions

Exogenous lactate causes an immediate decrease in glycolysis and increase in oxidative phosphorylation in resting human macrophages. The presence of lactate blocks the early Warburg effect in macrophages stimulated with Mtb or LPS. Furthermore, lactate reduced IL-1β and TNF production and increased bacillary killing in human macrophages infected with Mtb.

Put in the context of Mtb infection *in vivo*, we deduce that an accumulation of lactate in the extracellular milieu, caused by infected macrophages, has an immediate negative feedback effect on bystander unstimulated macrophages; causing them to downregulate glycolysis and upregulate oxidative phosphorylation. The reasons for this could be to limit inappropriate pathological inflammation and promote resolution or it may also serve to prevent resting macrophages competing for glucose resources when in the presence of other highly glycolytic cells. Since this anti-inflammatory effect is also, somewhat counterintuitively, associated with increased ability to kill the bacteria, we postulate that the previously described association between glycolysis and bacillary clearance may, at least in part, be mediated by the effects of lactate, the end-product of glycolysis (3–5).

These data indicate that aerosolised lactate delivered to the lungs may hold potential as a host-directed therapy for active pulmonary TB, where there is both ongoing mycobacterial replication and destruction of pulmonary tissue due to an unchecked pro-inflammatory response. Mtb blocks autophagic flux as a method of subverting host defence (50, 51). Our data indicates that lactate promotes autophagy which contributes to increased Mtb killing in human MDM. Autophagy also plays a role in a wide range of other disease states such as Crohn’s disease, cancer and heart disease (52), indicating that lactate may hold therapeutic potential in many disease settings. Accordingly, the precise mechanistic processes and molecular mediators through which lactate promotes autophagy to enhance Mtb killing warrants further elucidation in primary human and murine models to support the development of novel targeted therapies.

## Data Availability Statement

The raw data supporting the conclusions of this article will be made available by the authors, without undue reservation.

## Ethics Statement

The studies involving human participants were reviewed and approved by Research Ethics Committee School of Medicine, Trinity College Dublin. The patients/participants provided their written informed consent to participate in this study.

## Author Contributions

CÓM: conceptualisation, methodology, formal analysis, investigation, writing- original draft, writing- review and editing, visualisation, project administration and funding acquisition. DC: methodology, formal analysis, investigation, visualisation, writing -review and editing. JP: investigation, writing -review and editing. MM: investigation, writing -review and editing. DM: investigation, writing -review and editing. GL: investigation, writing -review and editing. LT: investigation, writing -review and editing. SO’L: investigation, writing -review and editing. KG: investigation, project administration, writing -review and editing. KMQ: investigation, writing -review and editing. AC: investigation, writing -review and editing. SG: conceptualisation, writing -review and editing and supervision. SB: conceptualisation, formal analysis, writing -original draft, writing -review and editing, visualisation, supervision and funding acquisition. JK: conceptualisation, writing -review and editing, supervision and funding acquisition. All authors contributed to the article and approved the submitted version.

## Funding

This work was funded by The National Children’s Research Centre (Grant D/18/1), The Royal City of Dublin Hospital Trust, Science Foundation Ireland award SFI/15/IA/3154 and the Health Research Board EIA-2019-010.

## Conflict of Interest

The authors declare that the research was conducted in the absence of any commercial or financial relationships that could be construed as a potential conflict of interest.

## Publisher’s Note

All claims expressed in this article are solely those of the authors and do not necessarily represent those of their affiliated organizations, or those of the publisher, the editors and the reviewers. Any product that may be evaluated in this article, or claim that may be made by its manufacturer, is not guaranteed or endorsed by the publisher.
