# Supplementary material for: Lactate Alters Metabolism in Human Macrophages and Improves Their Ability to Kill Mycobacterium tuberculosis
Source: Front Immunol. 2021 Oct 6;12:663695. doi: 10.3389/fimmu.2021.663695 (PMC8526932; doi:10.3389/fimmu.2021.663695)
Supplement: Supplementary file 1 [file DataSheet_1.docx]

**Supplementary Figure 1. MDM viability after the addition of lactate.** PBMC were isolated from buffy coats and MDM were adherence purified for 7 days in 10% human serum. The cells were treated with 0, 6.25, 12.5, 25, 50 and 100 mM of lactate three hours prior to infection with Mtb (iH37Rv or H37Ra), at an MOI of approximately 70% infectivity, 1-10 mycobacteria per cell, or stimulation with LPS (100 ng/ml). At 24 hours (A), 48 hours (B) and 120 hours (C) cells were stained with Hoechst and PI and total number of live cells was determined using the Cytell Cell Imaging System. (n=3 ±SD) Statistical significance was determined using two-way ANOVA with Tukey multiple comparisons test; *****P*<0.0001, ****P*<0.001, ***P*<0.01, **P*<0.05.

**Supplementary Figure 2. NaCl does not induce metabolic changes in human MDM.** PBMC were isolated from buffy coats and MDM were adherence purified for 7 days in 10% human serum. For A&B, MDM were gently scraped and seeded on Seahorse culture plates prior to analysis in the Seahorse XFe24 Analyzer. Differences in cell density was corrected for by % comparison to the basal ECAR and OCR. The ECAR and OCR were recorded approximately every 9 minutes. After 30 minutes, the Seahorse Analyzer injected NaCl (25 mM) into assigned wells. The ECAR and OCR readings were then continually sampled in real time. The time-course graphs illustrate the ECAR and OCR (A) of human MDM in real-time in response to treatment with NaCl. At the time point indicated, 40 mins after administration, the ECAR/OCR ratio (B) was calculated (n=4 ± SD). For C, MDM were left untreated or treated with 6.25, 12.5 and 25 mM of NaCl for three hours prior to infection with Mtb (H37Ra) at a MOI of 30-40% infectivity, 1-5 mycobacteria per cell (as determined by auramine O staining) and were then lysed on day 5 and plated on Middlebrook 7H10 agar (+OADC). CFU were enumerated 21 days after plating. Statistical significance was determined using a paired Student’s t-test (B) or one-way ANOVA with Tukey multiple comparisons test (C; n=3 ± SD).

**Supplementary Figure 3. Metabolic substrate dependency in human MDM.** PBMC were isolated from buffy coats and MDM were adherence purified for 7 days in 10% human serum (n=3). MDM were gently scraped and seeded on Seahorse culture plates. Cells were treated with lactate for 3 hours prior to stimulation with iH37Rv at an MOI of approximately 70% infectivity, 1-10 mycobacteria per cell for 24 hours before performing the Mito Fuel Flex Test on the Seahorse XFe24 Analyzer. Statistical significance was determined using two-way ANOVA with Sidak’s multiple comparisons test; ***P*<0.01.

**Supplementary figure 4: Cell surface expression of markers associated with M1/M2 phenotype and function.** PBMC were isolated from buffy coats and MDM were adherence purified for 7 days in 10% human serum (n=4). MDM were treated with lactate (25 mM) for 3 hours prior to stimulation with Mtb (MOI 1-10, 70% infectivity; extracellular bacteria were washed away after 3 hours) or LPS (100 ng/ml). 24 hours post stimulation, cells were washed, placed in ice cold PBS for 30 minutes to detach and placed in flow cytometry tubes. Cells were Fc blocked, stained with viability dye zombie NIR and stained with fluorochrome conjugated antibodies specific for CD14, CD68, CD40, HLA-DR, MMR, CD83, CD80 and CD86. Cells were acquired on a BD FACS Canto II and analysed using flowjo software. Unstained and fluorescence minus one controls were used to set gates. (A) The gating strategy for MDM. (B) Representative histograms showing the median fluorescence intensity (MFI) for CD40; left, and collated data (right). (C) Representative histograms showing HLA-DR expression, left, and collated data, right. (D) Representative histograms showing MMR expression, left, and collated data, right. (E) Representative histograms showing CD83 expression, left, and collated data, right. (F) Representative histograms showing CD80 expression, left, and collated data, right. (G) Representative histograms showing CD86 expression, left, and collated data, right. Data were analysed by two-way ANOVA; however, results were not statistically significant.

**Supplementary Figure 5. Lactate does not significantly alter metabolic function or cytokine production in human MDM 5 days post stimulation.** PBMC were isolated from buffy coats and MDM were adherence purified for 7 days in 10% human serum. (A) Cells were treated with lactate (25 mM) for 3 hours prior to infection with Mtb (H37Ra; MOI 1-5, 30-40% infectivity). After 24 hours, cells were lysed and the presence of LC3-II, p62 and β-actin were determined by Western blotting. All protein bands were normalized to their own β-actin controls and the expression of LC3-II or p62 were determined relative to the untreated, uninfected control (A; n=2). (B) Western blot images of 2 other donors, to support the representative image in main text figure 4. (C) Western blot image showing the effects of a titration of lactate concentrations on the expression of LC3-I, LC3-II and p62 in MDM infected with Mtb compared with controls. (D) MDM were gently scraped post differentiation and seeded on Seahorse culture plates prior to stimulation with iH37Rv (at an MOI of approximately 70% infectivity, 1-10 mycobacteria per cell) or LPS (100 ng/ml). On day 5 post stimulation, ECAR and OCR were determined on the Seahorse XFe24 Analyzer and the ECAR/OCR ratio was calculated based on the third baseline reading (D; n=3). The concentrations of TNF and IL-10 present in the supernatants were determined by ELISA 5 days post stimulation with iH37Rv (C). Each data point represents an individual donor (n=3-4). Statistical significance was determined using two-way ANOVA with Sidak’s multiple comparisons test, however; results were not statistically significant.
